# Relationship of tumor size with pathological and prognostic factors for hilar cholangiocarcinoma

**DOI:** 10.18632/oncotarget.22054

**Published:** 2017-10-23

**Authors:** Hai-Jie Hu, Rong-Xing Zhou, Anuj Shrestha, Yong-Qiong Tan, Wen-Jie Ma, Qin Yang, Jiong Lu, Jun-Ke Wang, Yong Zhou, Fu-Yu Li

**Affiliations:** ^1^ Department of Biliary Surgery, West China Hospital of Sichuan University, Chengdu, Sichuan Province, China; ^2^ Department of General Surgery, Gandaki Medical College, Pokhara, Nepal

**Keywords:** hilar cholangiocarcinoma, prognostic factors, DeOliveira staging system, tumor size, survival outcome

## Abstract

**Objective:**

To determine the correlation of different tumor-size cutoffs with prognostic factors and survival outcomes to provide a reference for the modification of the T-stage classification in the DeOliveira staging system for hilar cholangiocarcinoma (HCCA).

**Materials and Methods:**

We retrospectively analyzed 216 patients who underwent curative surgery for HCCA (mean tumor diameter, 2.8 cm) between 2000 and 2013. Univariate and multivariate logistic regression were used to assess the correlation of tumor-size cutoffs with various factors.

**Results:**

Tumor differentiation (odds ratio [OR]: 1.649, 95% confidence interval [CI]: 1.065–2.555, *P* = 0.025), node status (OR: 1.971, 95% CI: 1.060–3.664, *P* = 0.032), resection margin (OR: 2.465, 95% CI: 1.024–5.937, *P* = 0.044), and hepatectomy (OR: 2.373, 95% CI: 1.226–4.593, *P* = 0.01) were independently correlated with the 2-cm cutoff, while tumor differentiation (OR: 1.755, 95% CI: 1.062–2.091, *P* = 0.028), node status (OR: 2.166, 95% CI: 1.054–4.452, *P* = 0.035), and tumor margin (OR: 2.539, 95% CI: 1.089–5.919, *P* = 0.031) were independently associated with the 3-cm cutoff.

**Conclusions:**

The 2-cm and 3-cm cutoffs were strongly correlated with resection margin, node status, tumor differentiation and survival. The 2-cm cutoff may be added to the DeOliveira staging system.

## INTRODUCTION

Hilar cholangiocarcinoma (HCCA) is a comparatively infrequent neoplasm typically involving the confluence of the hepatic ducts [1]. Owing to their location at the confluence of the right and left bile ducts and their close relationship with major vascular structures, these tumors tend to invade the portal vein, hepatic artery, and liver parenchyma, resulting in a relatively low surgical-resectability rate of 34%–47.2% [2–4]. In addition, risk factors like preoperative liver dysfunction, obstructive jaundice, and long operative time increase the surgical risks and decrease the surgical-resection and survival rates. Despite this, the standard treatment for HCCA is bile-duct resection accompanied with hepatectomy, caudate lobectomy, lymphadenectomy, and even vascular resection and reconstruction [5–9]. HCCA-resection surgery is one of the most difficult and challenging surgical procedures performed by hepatobiliary surgeons because HCCA demonstrates a propensity for intramural growth, perineural invasion, and regional and distant lymphatic infiltration [8, 10]. The reported 5-year survival rates vary from 10% to 40%, which is far from satisfactory [1, 11–19].

Many prognostic factors such as lymph node status, tumor differentiation, margin status, and caudate lobectomy have been reported to influence survival outcomes in HCCA [6, 15, 20–23]. Tumor size is a controversial prognosticator; several studies have reported no correlation of tumor size with survival [4, 11, 24–26], while others have found that smaller tumors are associated with better survival results than larger tumors [22, 27–30]. In fact, the DeOliveira staging system for HCCA distinguishes between T1, T2, and T3 tumors solely on the basis of tumor diameter (< 1 cm vs. 1–3 cm vs. > 3 cm) [29, 31].

The DeOliveira staging system is now accepted as a classification system for HCCA. However, the practical ability of the staging system to predict the nature of the tumor or the postoperative survival outcomes remains to be further improved [31, 32]. In addition, few studies have specifically analyzed the correlation of tumor size with pre- and postoperative prognostic factors, and the influence of tumor size on clinical and pathological variables remains unclear. Actually, any staging system including the DeOliveira staging system, is undoubtedly not so comprehensive, thus further research is required to determine the relationship of different tumor sizes with various tumor factors in order to provide a reliable basis for the future definition of the T stage in the DeOliveira staging system.

We, therefore, conducted a single-center large-scale case series study that aimed to determine the relationship of different tumor-size cutoff points with various clinical and pathological variables as well as survival rates in order to evaluate the possibility of implementing another tumor-size cutoff of 2 cm or 4 cm in addition to the current 1-cm and 3-cm cutoff points of the DeOliveira staging system.

## RESULTS

### Patient characteristics

The study involved 216 HCCA patients, including 140 men and 76 women, with a median age of 60 years (range, 26–82 years). The clinical characteristics of the patients are shown in Table 1. The most common symptom was jaundice (60%). The median intraoperative blood loss was 600 mL (range, 50–2000 mL), and 103 (47.7%) patients required perioperative blood transfusion. Preoperative biliary drainage was performed in 107 (49.5%) patients with jaundice, and 30 (13.9%) patients underwent preoperative portal vein embolization. According to the AJCC staging system, 34, 76, 81, and 25 patients had stage I, II, III, and IV disease, respectively. According to the Bismuth-Corlette classification, 55, 52, 57, and 52 patients had type I, II, III, and IV disease, respectively. The perioperative morbidity rate was 31.9% (*n* = 69), and the most common perioperative complication was bile leakage (*n* = 25). The perioperative mortality rate was 2.3% (*n* = 5), which was defined as death within 60 d of the surgery, or occurred at any time during the postoperative hospital stay.

**Table 1:** Patient characteristics

Patient Characteristics (*n* = 216)	
Variables	Number (%) or median [range]
Age	60 [26–82]
Gender, male	140 (64.8)
Preoperative laboratory data	
Preoperative CA 19-9 level, U/ml	348 [0.6–1000]
Preoperative CA 125 level, U/ml	19.84 [1.23–257.7]
Preoperative CEA level, ng/ml	3.23 [0.2–65.51]
Preoperative TB level, mg/dL	10.2 [0.41–34.3]
Preoperative ALT level, U/L	95 [10–967]
Preoperative AST level, U/L	86 [14–1016]
Preoperative Albumin level, g/L	36.7 [18.7–51.8]
Preoperative hospital stay (median [range])	8 [2–44]
Total hospital stay (median [range])	19 [9–113]
Estimated blood loss, median [range]	600 [50–2000]
Tumor extent (Bismuth-Corlette)	
Type I	55 (25.4)
Type II	52 (24.1)
Type IIIa or IIIb	57 (26.4)
Type IV	52 (24.1)
AJCC T Stage	
T1	34 (15.7)
T2	76 (35.2)
T3	81 (37.5)
T4	25 (11.6)
DeOliveira T Stage	
T1 (Tumor size ≤ 1cm)	21 (9.7)
T2 (Tumor size 1-3 cm)	145 (67.1)
T3 (Tumor size > 3 cm)	50 (23.1)

CA19-9: carbohydrate antigenic determinant 19-9; CA125: carbohydrate antigen 125; CEA: Carcino Embryonie Antigen; TB: total bilirubin; ALT: alanine aminotransferase. AST: aspartate transaminase; AJCC: American Joint Committee On Cancer.

### Histopathology

On the postoperative pathological examination, all tumors were identified as adenocarcinomas. The tumor diameters ranged from 1 cm to 8 cm (median, 2.8 cm). With regard to the gross features, the most common phenotype was infiltrating tumor (*n* = 145, 67.1%), followed by nodular tumor (*n* = 51, 23.6%) and papillary tumor (*n* = 20, 9.3%). The margin status was R0 in 176 (81.5%) patients and R1 in 40 (18.5%) patients. In terms of tumor differentiation, 61 (28.2%) tumors were well differentiated, 99 (45.8%) were moderately differentiated, and 56 (26.0%) were poorly differentiated. Histologically negative lymph nodes were found in 56% (121/216) of patients.

### Survival

**Figure 1:**
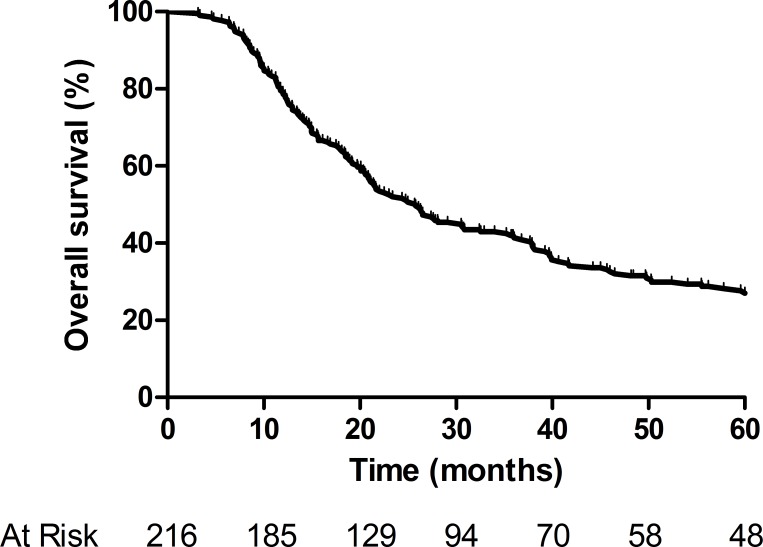
Overall survival of patients who underwent curative surgery for hilar cholangiocarcinoma

The median follow-up time was 21 months (range, 3–85 months). After resection, the median overall survival time was 26 months, and the 1-, 3-, and 5-year survival rates were 79%, 42%, and 27%, respectively (Figure 1). The median disease-free survival time was 17 months, and the 1-, 3-, and 5-year disease-free survival rates were 72%, 20%, and 11%, respectively. The factors that could predict survival outcomes are presented in Table 2. Among these factors, the 2-cm and 3-cm tumor-size cutoffs were independently associated with survival in both the univariate and multivariate analyses, while the 1-cm and 4-cm cutoffs failed to predict survival (*P* > 0.05).

**Table 2:** Univariate and multivariate analysis of factors associated with long-term survival after resection of hilar cholangiocarcinoma

Prognostic factors *P* value		Univariate analysis			Multivariate analysis	
		Hazard ratio	95% CI	*P* value	Hazard ratio	95% CI
Tumor size						
≤ 2 cm	Reference				Reference	
2–3 cm	0.045	1.406	1.008–1.959	0.007	1.596	1.135–2.244
> 3 cm	< 0.001	2.107	1.442–3.079	0.009	1.700	1.140–2.535
Positive nodal status	< 0.001	1.884	1.410–2.517	0.002	1.602	1.184–2.169
Poor differentiation	< 0.001	2.887	1.950–4.275	< 0.001	2.449	1.671–3.738
Positive resection margin	< 0.001	2.490	1.724–3.595	< 0.001	2.266	1.513–3.393

Additional factors not significant on univariate analysis included age, gender, the 1 cm and 4 cm tumor size cut-off, AJCC tumor stage, gross feature et al.

### Relationship between tumor size and other tumor characteristics

The relationship of the 2-cm and 3-cm tumor-size cutoffs with various tumor characteristics is presented in Table 3. The univariate logistic regression showed that the 2-cm cutoff was significantly correlated with tumor differentiation (*P* = 0.008), tumor-free resection (*P* = 0.009), node status (*P* = 0.001), preoperative hospital stay (*P* = 0.009), total hospital stay (*P* = 0.002), vascular invasion (*P* = 0.036), caudate lobectomy (*P* = 0.047), hepatectomy (*P* = 0.046), the AJCC T stage (*P* = 0.005), and the Bismuth-Corlette classification (*P* = 0.04). Similarly, the univariate logistic regression also showed that tumor differentiation (*P* < 0.001), tumor-free resection (*P* = 0.005), node status (*P* = 0.002), gross features (*P* = 0.021), preoperative hospital stay (*P* = 0.001), total hospital stay (*P* = 0.003), and vascular invasion (*P* = 0.021) were independently associated with the 3-cm tumor-size cutoff. However, in the subgroup of patients with tumors measuring *≤* 2 cm, no factors were identified to correlate with the 1-cm cutoff (*P* > 0.05). Similarly, no factors were associated with the 4-cm cutoff in the subgroup of patients with tumors measuring > 3 cm (*P* > 0.05).

**Table 3:** Pathological and prognostic risk factors correlated with tumor size

	Tumor Size (*n* = 216)				
Variable	≤ 2 cm (*n* = 88)	2-3 cm (*n* = 78)	P^*^	> 3 cm (*n* = 50)	P^**^
Age					
≤ 60	43 (48.9)	39 (50.0)	NS	26 (52.0)	NS
> 60	45 (51.1)	39 (50.0)		24 (48.0)	
Gender					
male	61 (69.3)	49 (62.8)	NS	30 (60.0)	NS
female	27 (30.7)	29 (37.2)		20 (40.0)	
Preoperative hospital stay					
≤ 8	69 (78.4)	50 (64.1)	0.009	23 (46.0)	0.001
> 8	19 (21.6)	28 (35.9)		27 (54.0)	
Total hospital stay					
≤ 19	59 (67.0)	41 (52.6)	0.002	16(32.0)	0.003
> 19	29 (33.0)	37 (47.4)		34(68.0)	
Surgical procedures					
BDR	20 (22.7)	10 (12.8)	0.046	6 (12.0)	NS
BDR+hepatectomy	68 (77.3)	68 (87.2)		44 (88.0)	
Caudate lobectomy					
Yes	72 (81.8)	70 (89.7)	0.047	45 (90.0)	NS
No	16 (18.2)	8 (10.3)		5 (10.0)	
Bismuth-Corlette classification					
I and II	51 (58.0)	31 (39.7)	0.04	25 (50.0)	NS
III and IV	37 (42.0)	47 (60.3)		25 (50.0)	
Gross feature					
Infiltrating tumor	58 (65.9)	57 (73.1)	NS	30 (60.0)	0.021
Nodular tumor	20 (22.7)	13 (16.7)		18 (36.0)	
Papillary tumor	10 (11.4)	8 (10.2)		2 (14.0)	
Differentiation					
Poor	13 (14.8)	19 (24.4)	0.008	24 (48.0)	< 0.001
Moderate	47 (53.4)	37 (47.4)		15 (30.0)	
Well	28 (31.8)	22 (28.2)		11 (22.0)	
Resection margin					
Positive	8 (9.1)	12 (15.4)	0.009	20 (40.0)	0.005
Negative	80 (90.9)	66 (84.6)		30 (60.0)	
lymph node metastasis					
Yes	27 (30.7)	33 (42.3)	0.001	35 (70.0)	0.002
No	61 (69.3)	45 (57.7)		15 (30.0)	
T stage (AJCC)					
T1 /T2	55 (62.5)	34 (43.6)	0.005	21 (42.0)	NS
T3 /T4	33 (37.5)	44 (56.4)		29 (58.0)	
Vascular invasion					
Yes	6 (6.9)	10 (12.8)	0.036	11 (22.0)	0.021
No	82 (93.1)	68 (87.2)		39 (88.0)	

P^*^: comparison between tumor size ≤ 2 cm versus tumor size > 2 cm; P^**^: comparison between tumor size ≤ 3 cm versus tumor size > 3 cm; CA19-9: carbohydrate antigenic determinant 19-9; BDR: Bile ducts resection alone; AJCC: American Joint Committee on Cancer.

Factors with *p* values < 0.1 from the univariate analysis were entered into in the multivariate analysis (Table 4). The multivariate analysis showed that tumor differentiation (odds ratio [OR]: 1.649, 95% confidence interval [CI]: 1.065–2.555, *P* = 0.025), node status (OR: 1.971, 95% CI: 1.060–3.664, *P* = 0.032), tumor resection margin (OR: 2.465, 95% CI: 1.024–5.937, *P* = 0.044), hepatectomy (OR: 2.373, 95% CI: 1.226–4.593, *P* = 0.01), and total hospital stay (OR: 2.428, 95% CI: 1.327–4.441, *P* = 0.004) were independently associated with the 2-cm cutoff. However, other factors like preoperative hospital stay (OR: 1.671, 95% CI: 0.798–3.489, *P* = 0.17), vascular invasion (OR: 0.796, 95% CI: 0.274–2.313, *P* = 0.67), the AJCC T stage (OR: 1.585, 95% CI: 0.829–3.030, *P* = 0.16), the Bismuth-Corlette classification (OR: 1.154, 95% CI: 0.544–2.451, *P* = 0.79), and caudate lobectomy (OR: 1.649, 95% CI: 0.780–3.484, *P* = 0.19) failed to maintain a significant statistical difference in the multivariate analysis. In addition, the multivariate analysis showed that tumor differentiation (OR: 1.755, 95% CI: 1.062–2.091, *P* = 0.028), node status (OR: 2.166, 95% CI: 1.054–4.452, *P* = 0.035), tumor resection margin (OR: 2.539, 95% CI: 1.089–5.919, *P* = 0.031), and total hospital stay (OR: 2.383, 95% CI: 1.072–5.295, *P* = 0.033) were independently associated with the 3-cm cutoff. However, gross pathological features (OR: 0.773, 95% CI: 0.427–1.399, *P* = 0.394), preoperative hospital stay (OR: 1.980, 95% CI: 0.890–4.403, *P* = 0.094), and vascular invasion (OR: 0.519, 95% CI: 0.198–1.359, *P* = 0.182) were not significantly associated with the 3-cm cutoff in the multivariate analysis.

**Table 4:** Variables associated with tumor size in a multivariate logistic analysis

Variables	2 cm cut-off			3 cm cut-off		
	Odds ratio	95% CI	*P* value	Odds ratio	95% CI	*P* value
Tumor resection margin	2.465	1.024–5.937	0.044	2.539	1.089–5.919	0.031
Tumor differentiation	1.649	1.065–2.555	0.025	1.755	1.062–2.091	0.028
Node status	1.971	1.060–3.664	0.032	2.166	1.054–4.452	0.035
Total hospital stay	2.428	1.327–4.441	0.004	2.383	1.072–5.295	0.033
Hepatectomy	2.373	1.226–4.593	0.010	-	-	-

CI: confidence interval

## DISCUSSION

HCCA is a highly fatal disease. Despite rapid developments in surgical techniques and perioperative care, the resection of HCCA remains challenging. The prognostic factors for HCCA have been extensively researched. However, to our knowledge, the relationship of different tumor sizes with prognostic factors such as pathological classification, nodal status, resection margins and survival, has not yet been investigated in detail. Moreover, different authors hold different views about the prognostic effects of tumor size and the T stage of the DeOliveira staging system, and whether or not 2-cm and 4-cm cutoffs should be included in the T stage of the DeOliveira staging system remains debatable. Some studies have reported that a tumor size of ≥ 2 cm is associated with worse survival outcomes [22, 33]. Therefore, in the present study, we assessed tumor-size cutoffs of 1, 2, 3, and 4 cm, so as to determine the relationship of tumor size with various prognostic factors and survival outcomes. Our study comprises a single-center large-scale case series of HCCA patients treated over a period of 10 years. Thus, it is the first large-scale case series to specifically evaluate the relationship of different tumor-size cutoffs with pathological and prognostic factors in a large sample of HCCA cases. We hope that our results will provide a basis for the reevaluation of the T stage in the DeOliveira staging system.

The specific analysis of the relationship of different tumor-size cutoffs with tumor differentiation showed that the 2-cm and 3-cm cutoffs were correlated with tumor differentiation, with larger tumors being more likely to be poorly differentiated. However, the 1-cm and 4-cm cutoffs showed no correlation with tumor differentiation. Tumor differentiation has been reported in the literature as a measure of the biological aggressiveness of a tumor and a predictor of long-term survival [15, 17, 24, 34]. Therefore, considering the finding that the 2-cm and 3-cm tumor size cutoffs were associated with tumor differentiation, it is unsurprising that they also predicted the overall survival. Thus, we consider that besides the current tumor-size cutoffs of 1 and 3 cm, the 2-cm cutoff may also be included in the DeOliveira staging system.

A detailed analysis of the correlation between tumor-size cutoffs and lymph node metastasis highlighted the intimate relationship between tumor diameter and lymph node metastasis. Compared with 1-cm cutoff, the 2-cm and 3-cm cutoffs were indeed associated with a higher proportion of lymph node metastasis. Node status has been proved to be an independent factor influencing the overall outcome in many recent research studies [35, 36]. Thus, our results indicate that the 2-cm and 3-cm tumor-size cutoffs can indirectly affect the overall survival owing to their correlation with node status. We consider that the 2-cm cutoff may become a new potential tumor-size cutoff point in the DeOliveira staging system in future.

We further evaluated the relationship of different tumor-size cutoffs with resection margins. The 2-cm and 3-cm cutoffs were correlated with microscopically negative surgical margins. Specifically, the likelihood of achieving a microscopically negative margin decreased from 90.9% to 60% as the tumor size increased from 2 cm to 3 cm or larger. However, in the subgroups of patients with tumors measuring *≤* 2 cm and those measuring > 3 cm, we did not find any correlation between tumor size and resection margins. Thus, compared with 1-cm cutoff, the 2-cm and 3-cm cutoffs were strongly correlated with the resection margin. Furthermore, the importance of R0 resection margins in prolonging the overall survival rate has been frequently reported by many authors [27, 37, 38]. Our results showed that the 2-cm and 3-cm tumor-size cutoffs were strongly associated with resection margins and thus indirectly influenced postoperative survival outcomes to some extent. This finding further supports the addition of a 2-cm cutoff to the DeOliveira staging system.

Upon analyzing the relationship of different tumor-size cutoffs with surgical procedures, we found that the 2-cm cutoff was correlated with whether to carry out hepatectomy or not. Hepatectomy appears to have a positive effect on tumor-free margins and survival after the resection of Klatskin tumors [23, 39]. Thus, the 2-cm cutoff may be more important than the 3-cm cutoff because of its additional impact on the application of hepatectomy and indirect effect on survival outcomes. We therefore recommend that the 2-cm cutoff be included in the DeOliveira staging system.

Consistent with the current DeOliveira staging system, our results indicated that the 3-cm cutoff was a significant prognostic factor and that this cutoff point was reasonable for HCCA, as tumors larger than 3 cm were more likely to be associated with a poor prognosis [29, 30]. However, different from the existing DeOliveira staging system, our results showed that in addition to the 3-cm tumor-size cutoff, the 2-cm cutoff was also correlated with tumor differentiation, node status, and resection margins and indirectly affected long-term survival. Furthermore, it could affect the ability to carry out surgical procedures and further influence survival outcomes. In the current DeOliveira staging system, the T stage is classified according to tumor size into the TI (< 1 cm), T2 (1–3 cm), and T3 (> 3 cm) stages, which ignores the role of the 2-cm tumor-size cutoff. Thus, our findings might complement the current DeOliveira staging system, and the 2-cm cutoff may become another potential tumor-size cutoff in the new DeOliveira staging system.

Also, different from the existing staging system, our study did not find an association of the 1-cm cutoff with prognostic factors and survival. In their research, DeOliveira et al. only interpreted the reason for selecting the 3-cm cutoff for defining T3 tumors; they did not explain why the 1-cm cutoff was selected as the first cutoff point [31]. Because of the late presentation of symptoms in HCCA, these tumors are usually diagnosed in the later stages when the disease is locally advanced. Thus, most patients have tumors measuring > 1 cm, which is supported by our current findings. So this classification needs to be further evaluated in a large case series or multi-center clinical research study.

In conclusion, our data confirm the notion that compared with the 1-cm tumor-size cutoff, the 2-cm and 3-cm cutoffs were more important in terms of their association with survival and other prognostic factors. Compared with patients with tumors measuring *≤* 2 cm, those with tumors measuring > 3 cm were more likely to have poorly differentiated tumors, R1 or R2 resection margins, and lymph node metastasis. The 2-cm and 3-cm tumor-size cutoffs could also affect the overall survival outcome by indirectly influencing tumor differentiation, tumor resection margins, and node status. The 2-cm and 3-cm tumor-size cutoffs maybe more reasonable than the current 1-cm and 3-cm cutoffs. At the very least, the 2-cm cutoff should be considered as another potential tumor-size cutoff point in the current DeOliveira staging system; however, further research is urgently warranted to clinically test the rationality of the present results.

## MATERIALS AND METHODS

### Study population and data collection

Patients with pathologically verified HCCA from January 2000 to January 2013 were retrospectively involved after curative resection and clinical, radiologic, pathologic, intraoperative and survival data were systematically analyzed. Periampullary carcinomas, gallbladder cancers, or intrahepatic cholangiocarcinomas and those who underwent palliative surgery were excluded from this study. Total of 216 patients were divided into three groups based on tumor size: A: ≤ 2 cm (*n* = 88), B: 2-3 cm (*n* = 78), C: > 3 cm (*n* = 50). Tumor size ≤ 2 cm was further divided into two subgroups: tumor size ≤ 1 cm (*n* = 21) and tumor size 1-2 cm (*n* = 67). Similarly, tumor size > 3 cm was also divided into two subgroups: tumor size 3-4 cm (*n* = 39) and tumor size > 4 cm (*n* = 11).

### Preoperative evaluation

Patients were evaluated with a baseline of medical history, detailed physical examination, full blood count, liver function tests, serum carcinoembryonic antigen (CEA) and cancer antigen 19-9 (CA19-9) assays. Abdominal ultrasonography and abdominothoracic computed tomography were systematically performed to evaluate local and distant tumor involvement. Magnetic Resonance Cholangiopancreatography was used in patients who were considered to have potentially resectable tumors. Preoperative biliary decompression was performed in patients with cholangitis or obstructive jaundice (> 5 mg/dL) through percutaneous transhepatic biliary drainage or endoscopic biliary drainage. Portal vein embolization was mainly carried out on patients with compromised liver function when the anticipated future remnant volume was less than 25% of the total liver volume, in order to minimize postoperative hepatic failure risk. Resection procedures were selected according to preoperative imaging and intraoperative evaluation by experienced operating surgeons.

### Surgical procedures and tumor classification

**Figure 2:**
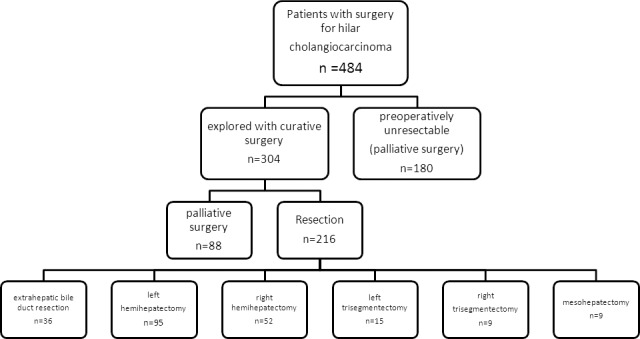
Flow diagram showing the results of surgical procedures of all patients with hilar cholangiocarcinoma in our series

The standard treatment as per our hospital guideline was biliary confluence resection combined with major hepatectomy and lymphadenectomy. The results of surgical procedures of patients with hilar cholangiocarcinoma are presented in Figure 2, of which portal vein resection and reconstruction was performed in 26 (12.0%) patients, en bloc caudate was routinely removed in 187 (86.6%) of these patients and patients that did not involve caudate lobe resection were early cases of type I papillary carcinoma. Intra-operative frozen section of resected bile ducts and liver parenchyma margin were routinely examined to guide resection. Biliary continuity was restored by Roux-en-Y choledocho-jejunostomy.

### Statistical analysis

Frequency and descriptive analysis were applied to describe patient characteristics. Univariate and bivariate analysis was used to discuss the association of several variables, including gender, age, CA199 level, preoperative biliary drainage, vascular invasion, surgical procedures, gross feature, Bismuth-Corlette classification, tumor staging (AJCC), tumor resection margin, tumor differentiation, node status and postoperative complications et al. with different cut-offs of tumor size. The significant factors (*P* < 0.10) identified by univariate analysis were included in a multivariate logistic regression to investigate the influence of these covariates on tumor size. The two-sided *P* values of < 0.05 were considered significant. Statistical analysis was performed using SPSS version16.0 (SPSS Inc, Chicago IL).

## Abbreviations

HCCA: Hilar cholangiocarcinoma
OR: odds ratio
CI: confidence interval
CA19-9: cancer antigen 19-9
